# Immunomodulatory Drugs Exert Anti-Leukemia Effects in Acute Myeloid Leukemia by Direct and Immunostimulatory Activities

**DOI:** 10.3389/fimmu.2018.00977

**Published:** 2018-05-04

**Authors:** Aude Le Roy, Thomas Prébet, Rémy Castellano, Armelle Goubard, Florence Riccardi, Cyril Fauriat, Samuel Granjeaud, Audrey Benyamine, Céline Castanier, Florence Orlanducci, Amira Ben Amara, Frédéric Pont, Jean-Jacques Fournié, Yves Collette, Jean-Louis Mege, Norbert Vey, Daniel Olive

**Affiliations:** ^1^Team Immunity and Cancer, Centre de Recherche en Cancérologie de Marseille (CRCM), INSERM, U1068, CNRS, UMR7258, Institut Paoli-Calmettes, Aix-Marseille University, UM 105, Marseille, France; ^2^Immunomonitoring platform, Institut Paoli-Calmettes, Marseille, France; ^3^Department of Internal Medicine, Section of Hematology, Yale University School of Medicine, New Haven, CT, United States; ^4^TrGET Platform, Centre de Recherche en Cancérologie de Marseille (CRCM), INSERM, U1068, CNRS, UMR7258, Institut Paoli-Calmettes, Aix-Marseille University, UM 105, Marseille, France; ^5^CiBi Platform, Centre de Recherche en Cancérologie de Marseille, Institut Paoli-Calmettes, INSERM, U1068, CNRS, UMR7258, Aix-Marseille Université UM 105, Marseille, France; ^6^Cancer Research Center of Toulouse (CRCT), UMR1037 INSERM/Université Toulouse III Paul Sabatier/ERL5294 CNRS, Oncopole de Toulouse, Toulouse, France; ^7^Microbes Evolution Phylogeny and infections (MEPHI), IHU Méditerranée Infection, Marseille, France; ^8^Hematology Department, Centre de Recherche en Cancérologie de Marseille (CRCM), INSERM, U1068, CNRS, UMR7258, Institut Paoli-Calmettes, Aix-Marseille University, UM 105, Marseille, France

**Keywords:** acute myeloid leukemia, immunomodulatory drugs, lenalidomide, natural killer cells, cereblon

## Abstract

Immunomodulatory drugs (IMiDs) are anticancer drugs with immunomodulatory, anti-angiogenesis, anti-proliferative, and pro-apoptotic properties. IMiDs are currently used for the treatment of multiple myeloma, myelodysplastic syndrome, and B-cell lymphoma; however, little is known about efficacy in acute myeloid leukemia (AML). We proposed in this study to investigate the relevance of IMiDs therapy for AML treatment. We evaluated the effect of IMiDs on primary AML blasts (*n* = 24), and the impact in natural killer (NK) cell-mediated immunosurveillance of AML. Using primary AML cells and an immunodeficient mouse leukemia xenograft model, we showed that IMiDs induce AML cell death *in vitro* and impair leukemia progression *in vivo*. In addition, treatment of AML blasts with IMiDs resulted in enhanced allogeneic NK cell anti-leukemia reactivity. Treatment by pomalidomide of AML blasts enhanced lysis, degranulation, and cytokine production by primary allogeneic NK cells. Furthermore, the treatment with lenalidomide of patients with myeloid malignancies resulted in NK cell phenotypic changes similar to those observed *in vitro*. IMiDs increased CD56 and decreased NKp30, NKp46, and KIR2D expression on NK cells. Finally, AML blasts treatment with IMiDs induced phenotypic alterations including downregulation of HLA-class I. The effect of pomalidomide was not correlated with cereblon expression and A/G polymorphism in AML cells. Our data revealed, a yet unobserved, dual effects on AML affecting both AML survival and their sensitivity to NK immunotherapy using IMiDs. Our study encourages continuing investigation for the use of IMiDs in AML, especially in combination with conventional therapy or immunotherapy strategies.

## Introduction

Thalidomide derivatives lenalidomide and pomalidomide are a class of oral anticancer agents known as immunomodulatory drugs (IMiDs). They are currently used in a variety of hematologic malignancies. Administration is approved in patients with transfusion-dependent anemia due to low risk myelodysplastic syndrome (MDS) with deletion of the long arm of chromosome 5 [del(5q)] (1), for the treatment of multiple myeloma (MM) (2) and for other B cell lymphomas (3, 4). IMiDs directly exert antitumor activity through different therapeutically relevant effects such as angiogenesis inhibition (5, 6), inhibition of cancer cell proliferation (7–9), and induction of apoptosis (10). Additionally, IMiDs have potent immunomodulatory effects. Hence, lenalidomide and pomalidomide stimulate T-cell proliferation, interleukin (IL)-2 and IFN-γ production by T cells (11–14). Many studies suggest that increased IL-2 production induced by IMiDs leads to natural killer (NK) cell activation, proliferation, and NK cell-mediated ADCC against tumor cells (6, 12, 15–18). Finally, direct effects of lenalidomide have been described by modification of NK cell phenotype without consequence on the cytotoxic potential of NK cells (19).

Immunomodulatory drugs molecular mechanisms are better defined since the identification of their molecular target, cereblon (CRBN), and the transcription factors IKZF1 and IKZF3 (20, 21). In particular, CRBN knockout on myeloma cell lines leads to lenalidomide and pomalidomide resistance (22, 23). Many recent studies reported also the role of CRBN in IMiDs response *in vitro* and *in vivo* in patients with MM, chronic lymphocytic leukemia, or MDS (9, 20–22, 24–27). However, to date, the presence of a deletion 5q with or without other chromosomal abnormalities is the best response prognosis factor of lenalidomide treatment in myeloid malignancies. Haploinsufficiency for the CDC25C, PP2A, and CSNK1A1 genes encoded within the common deleted region has been implicated in the pathogenesis of the del(5q) phenotype. Lenalidomide specifically targets haploinsufficient genes by inhibiting phosphatase activity of Cdc25C and PP2A, and by inducing degradation of CSNK1A1 by the CRBN-CRL4 E3 ubiquitin ligase complex (28–30).

Natural killer cells are anti-tumor innate lymphoid cells that play an important role in immunosurveillance of acute myeloid leukemia (AML). As demonstrated by clinical success of allogeneic stem cell transplantation, haploidentical transplantation with killer cell inhibitory receptor (KIR) ligand mismatch, adoptive transfer of allogeneic or autologous T cells or NK cells, peptide vaccination, and treatment with monoclonal antibodies, it is well demonstrated that the immune system, in particular NK cells, has a critical role in the control of AML initiation and progression. Furthermore, accumulating evidences highlight NK cell parameters as prognostic factors in AML patients (31–35). NK cells exert their anti-leukemic activity by direct killing of tumor cells through release of perforin and granzymes, and by death ligands. NK cell also secrete proinflammatory cytokines (such as IFN-γ and TNF-α) or chemokines (such as MIP-1 and RANTES) leading to activation of other immune cells. Activation of NK cell is finely tuned by a large array of activating or inhibitory receptors recognizing stress-induced ligands or adhesion molecules. Particularly, interaction of NK cells with leukemia cells is dependent on various molecules including ligands for activating (ligands for NKG2D, DNAM-1, and NCRs) and inhibitory [ligands for KIR and CD94/NKG2A (human leukocyte antigen class I molecules)] receptors (36–39). HLA-class I molecules expressed by tumor cells play a crucial role in the regulation of NK cell-mediated cytotoxicity. It has been postulated that NK cell avidly lyse tumor cells that do not display inhibitory KIR-ligand provided activating ligands were present. Moreover, the use of anti-HLA class I antibody in blocking experiment increases allogeneic NK cell lysis (40, 41) and targeting KIR-HLA-ABC or NKG2A/CD94-HLA-E interactions represent potential tools for immunotherapy against AML (42–46).

By their immunomodulatory effects, especially on T and NK cells, evaluation of IMiDs activity in AML is attractive. Few clinical trials or case reports have been conducted for lenalidomide in AML. Complete remission were achieved in del(5q) and in non-del(5q) AML patients treated with lenalidomide, alone or in combination with other agents (cytarabine, azacitidine) (47–50). To our knowledge, only one study has described lenalidomide effect *in vitro* on AML blasts without del(5q) and lymphocytes. Khaznadar et al. have shown that lenalidomide enhanced lytic granule polarization on AML cell lines and speculated that IMiDs could restore NK–AML synapses, therefore improving recognition of AML by NK cells (51).

We proposed here to investigate the relevance of IMiDs therapy for AML treatment. The aim of the study is to determine whether IMiDs are effective in the control of AML cell growth. We first studied the toxicity of IMiDs on primary AML cells *in vitro* and *in vivo* using a NSG (NOD-SCID IL-2Rγc deficient) mouse leukemia xenograft model. We next evaluated NK cell functions and NK cell capacity to lyse AML blasts pre-treated by IMiDs. Our data showed that IMiDs sensitized AML blasts to NK cell-mediated lysis. This effect was not associated with CRBN. Finally, IMiDs modulated NK receptor expression. We achieved an immunomonitoring study and showed that IMiDs induced similar effects on NK cell receptor expression *in vitro* and *in vivo*. IMiDs also modulated the expression of NK ligand on primary AML cells, and in particular, IMiDs induced the downregulation of HLA class I, the ligands for NK cell inhibitory receptors.

Herein, we report a novel mechanism of action of IMiDs and suggest that IMiDs can be used as immunomodulatory agents to sensitize AML cells to NK cell-mediated killing.

## Materials and Methods

### Reagents

Lenalidomide and pomalidomide are from Sigma-Aldrich. IMiDs were dissolved at the concentration of 80 mM in DMSO (Sigma-Aldrich) and stored at −80°C until further use. Except for dose-effect experiments, IMiDs were used at a final concentration of 10 µM. In all experiments, corresponding concentrations of DMSO were used as a negative control. Recombinant human (rh) IL-2 was purchased from Novartis, and rh-IL-15 from R&D systems.

### Patients

Twenty four patients from Institut Paoli Calmettes (Marseille, France) with non-del(5q) AML, entered this study after informed consent, obtained from all participants in accordance with the Declaration of Helsinki. Cells from peripheral blood were collected for twenty two of them at time of diagnosis, one at the moment of first relapse and one at the moment of evolution. The mononuclear cells (>75% of blasts) were isolated by density gradient centrifugation (Lymphoprep; AbCys) and cryopreserved until use in RPMI 1640 supplemented with 10% heat-inactivated FCS (Eurobio) containing 10% of DMSO (Sigma-Aldrich). Cells were cultured with IMiDs or control treatment for 48 h before used for *in vitro* cytotoxicity and flow cytometry experiments.

For the immunomonitoring study, six patients with myeloid malignancies treated with lenalidomide at the Institut Paoli-Calmettes were prospectively recruited between January 2012 and December 2013. The study number 2012-A01381-42 was undertaken in accordance with the principles of the Declaration of Helsinki and Good practice guidelines and after local ethics committee approval. Each patient gave written informed consent. The median age of patients was 69.5 (ranged 56–88). Five patients were treated with lenalidomide 10 mg, and one patient with 5 mg, daily on days 1–21 of repeated 28 day cycles. Blood were sampled at day 0 (D0), D15, and D28 of a month of treatment with lenalidomide. The mononuclear cells were isolated by density gradient centrifugation (Lymphoprep; AbCys) and cryopreserved until use. Phenotypying tests of NK cells were performed using multicolor flow cytometry.

### Cell Culture

Effector NK cells were established as follow. PBMCs from healthy volunteers (HV) were obtained from blood samples provided by the “Etablissement Français du Sang” (EFS, Marseille, France), after isolation by density gradient centrifugation (Lymphoprep; AbCys). NK cells were purified using a human NK Cell Isolation kit (EasySep; StemCell Technologies). NK cells were cultured overnight at the concentration of 4.10^6^ cells per mL in RPMI 1640 supplemented with 10% of heat-inactivated FCS, 100 IU/mL IL-2, and 10 ng/mL IL-15 at 37°C and 5% CO_2_. After overnight culture, NK cells were washed and used for *in vitro* experiments.

The erythroleukemia cell line K562 was obtained from the American Type Culture Collection and was cultured in complete RPMI 1640 medium with 10% heat-inactivated FCS. Cells in exponential growth phase were used for all assays.

### Flow Cytometry

Expression of ligands on primary AML cells, and NK cell receptors, was analyzed by flow cytometry. Cells were stained with fluorochrome-labeled mAbs according to the manufacturer’s instructions. Abs against CD45 (clone J.33, Beckman Coulter), in combination with anti-HLA-ABC (clone B9.12.1, Beckman Coulter), anti-HLA-E (clone 3D12HLA-E, eBioscience), anti-MICA-B (clone 6D4, BD Biosciences), anti-ULBP1 (clone 170818, R&D Systems), anti-Nectin-2 (CD112, clone R-2.477.1, Beckman Coulter), and anti-PVR (CD155, clone SK11.4, Biolegend) were used for AML cell phenotyping. Abs against CD56 (clone AF12-7H3, Miltenyi Biotec), CD3 (clone UCHT1, BD Biosciences), CD16 (clone 3G8, BD Biosciences), NKp30 (clone Z25, Beckman Coulter), NKp46 (clone 9E2, Miltenyi Biotec), NKp44 (clone Z231, Miltenyi Biotec), DNAM-1 (clone DX11, BD Biosciences), NKG2D (clone BAT221, Miltenyi Biotec), KIR2D (clone NKVFS1, Miltenyi Biotec), NKG2A (clone Z199, Beckman Coulter) were used for NK cell phenotyping. Control cells were stained with the corresponding isotype Abs and LIVE/DEAD Fixable Dead Cell Stain Kit was used for viability (Life Technologies). Following incubation and washing, samples were analyzed on an eight-color FACS Canto II flow cytometer using the DIVA software (Becton Dickinson, San Jose, CA, USA). AML blasts cells were gated using anti-CD45, side scatter plot. NK cells were gated as CD3^−^/CD56^+^ cells.

For analysis of CD107a expression, purified NK cells and target cells (cell ratio 1:1) were co-incubated at 37°C in the presence of soluble FITC-labeled CD107a mAbs (clone H4A3, BD Biosciences) conjugate and Golgistop^®^ (BD Biosciences). After 4 h, cells were collected, washed twice, and stained for surface markers [PERCPCy5.5-CD3 (clone UCHT1, BD Biosciences) and PECF594-CD56 (clone AF12-7H3, Miltenyi Biotec)]. For intracellular staining, cells were fixed and permeabilized (Cytofix/Cytoperm^®^, BD Biosciences) according to the manufacturer’s instructions, and stained with PE-conjugated anti-human IFN-γ (clone 45.15, Beckman Coulter) and APC-conjugated anti-human TNF-α (clone Mab11, BD Biosciences). Cells were finally re-suspended in PBS and analyzed on a BD FACS Canto II.

### Chromium Release Assay

*In vitro* cytotoxicity assays were performed with NK cells from HV against AML cells and the K562 cell line as positive control. Target cells (1.10^6^) were washed and incubated with 100 μCi [^51^Cr] (Perkin Elmer) for 90 min and mixed with effector cells at E:T ratios of 2:1 and 15:1 in 200 µL RPMI 1640 medium with 10% FCS. After 4 h incubation at 37°C and 5% CO_2_, 100 µL supernatant of each sample was transferred in Luma plates and radioactivity was determined with a gamma counter. The percentage of specific lysis was calculated using the standard formula [(experimental − spontaneous release)/(total − spontaneous release) × 100], expressed as the mean of triplicate samples and presented normalized to K562 lysis. In some cytotoxicity assays, we blocked the MHC-class I on target cells using anti-MHC class I (clone YJ4) or IgG1 control antibodies at final concentration of 10 µg/mL.

### Western Blot

For western blot, cells were washed twice with PBS, lysed by HNTG buffer (50 mM HEPES pH7, 150 mM NaCl, 10% Glycerol, 1% Triton-X, 1.5 mM MgCl_2_, 1 mM EGTA), and centrifuged for 10 min at 13,000 rpm at 4°C. Supernatants were kept at −80°C. 20 µg of total lysate were resolved by SDS-polyacrylamide gel electrophoresis (SDS-PAGE) in denaturing conditions, transferred onto PVDF membranes (Immobilon-P, Millipore), and then probed with anti-CRBN (clone ace-#44, kindly provided by Hiroshi Handa, Integrated Research Institute, Tokyo Institute of Technology, Yokohama 226-8503, Japan) and anti-GAPDH (clone mAbcam 9484, Abcam) antibodies. The bands were then visualized with HRP-conjugated anti-mouse IgG (Jackson Immunoresearch), and western blot chemiluminescence reagent (West Dura, Pierce). Signals were quantified using the ImageJ software. Data were then converted to a fold change ratio obtained by dividing the values for CRBN normalized with GAPDH.

### Quantitative RT-PCR

Quantitative RT-PCR analysis was performed with the Applied Biosystems 7900HT Fast Real-Time PCR system using Taqman detection according manufacturer’s instructions. Briefly, total RNA was isolated from AML cells using the standard TRIzol reagent protocol (Life Technologies). 2 µg of the obtained RNA was reverse transcribed using oligo(dT). Each PCR assay was performed in a 25 µl reaction containing 2× TaqMan universal Mix (Applied Biosystems) reagent, 20× primers and 2 µl cDNA (equivalent to 40 ng of total RNA). Ct (threshold cycle) was calculated for each assay (Sequence Detection System Software 2.4.1, Applied Biosystems). Data are normalized using GAPDH as endogenous control (ΔCt = Ct_CRBN_ − Ct_GAPDH_). Results are presented as *R* = 2^−ΔCt^. CRBN and GAPDH TaqMan Gene Expression assays were purchased from Applied Biosystems.

### CRBN Genotyping

DNA was isolated from PBMCs (>75% of blasts) from AML patients using Qiamp DNA Mini (QIAGEN) according to the manufacturer’s protocol. 20 ng of the obtained DNA was genotyped for A/G polymorphism located at −29 nt of the 5′-untranslated region of CRBN (located on chr3 at nt 3221430 and referenced as rs1672753) with Applied Biosystems 7900HT Fast Real-Time PCR system.

### NOG Mouse/Human AML Model

NOD-SCID IL-2Rγc deficient (NSG) mice were purchased from Charles River France or bred in-house and maintained under specific pathogen-free conditions. Mice maintenance and experimental procedures were performed in accordance with protocols approved and compliance with policies approved by the local Committee for Animal Experimentation of Marseille (CAE of Provence number 14), France (2-091009). Healthy 6- to 8-week-old male mice were sublethally irradiated (1.5 Gy) on D0 and injected i.v. with 1.10^6^ PBMCs from AML patient depleted for CD3^+^ T cells (depletion kit, MACS^®^ Technology, Miltenyi Biotec). When AML blasts were detected by cytometry in blood, mice were randomly assigned to receive four daily injections i.v. of pomalidomide (5 mg/kg in 100 µL of PBS) or control (PBS + DMSO). Progression of leukemia was evaluated in blood by flow cytometry using appropriated mAbs [anti-mouse CD45 (clone 30-F11, eBiosciences), anti-human CD45 (clone J.33, Beckman Coulter), anti-human CD33 (clone WM53, BD Biosciences)], and LIVE/DEAD Fixable Dead Cell Stain Kit for viability (Life Technologies). Number of hCD45^+^ cells per microliter of blood was quantified by flow cytometry using quantification beads (CountBright^TM^ absolute counting beads, Life Technologies).

### Statistics

Statistical analysis was performed using GraphPad Prism 5 software (GraphPad). Paired Student *t*-test (*n* > 30), Wilcoxon matched-pairs test (*n* < 30), and Mann–Whitney test (*n* < 30) were used where appropriate. A *p* value < 0.05 was considered statistically significant (*p < 0.05; **0.05 < *p* < 0.01; ***p < 0.001). Pearson correlation test was used to assess correlation between two sets of data.

## Results

### *In Vivo* and *In Vitro* Sensitivity of Primary AML Blasts to Pomalidomide

**Figure 1:**
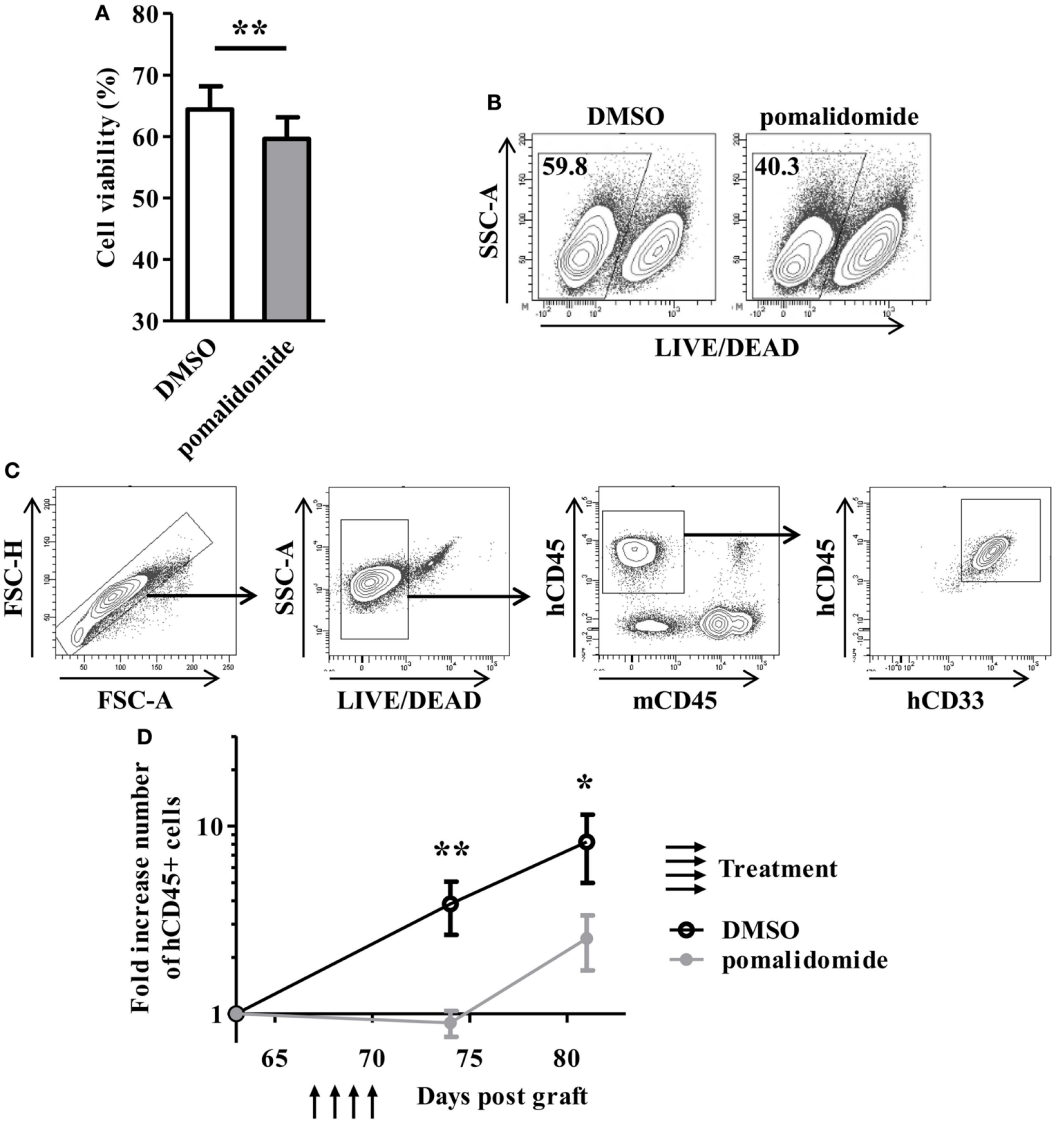
*In vitro* and *in vivo* exposure of acute myeloid leukemia (AML) blasts to pomalidomide induce tumor cell death and control leukemia growth. **(A,B)** Viable and death primary AML blasts were monitoring by flow cytometry using LIVE/DEAD discriminator staining. **(A)** Percentage of live cells after 48 h of culture with pomalidomide (10 µM) or DMSO control treatment (*n* = 23) were expressed as mean ± SEM and statistical significance was established using paired Student’s *t*-test. **(B)** Representative dot plot from one experiment (patient upn20). **(C,D)** NSG mice (*n* = 14) were irradiated on day 0 and i.v. inoculated with 1.10^6^ CD3-depleted primary AML cells (from patient upn20) on day 1. When AML blasts were detected by cytometry in blood (day 64 post graft), mice were randomly assigned to be i.v. injected with pomalidomide (5 mg/kg) (*n* = 7) or with DMSO (*n* = 7) on days 67, 68, 69, and 70. Both group have comparable leukemia burden at day 64 before treatment (mean ± SEM, 6.8 ± 3.6 cells per μL for DMSO group versus 7.4 ± 1.8 for pomalidomide group, *p* = 0.62) **(C)** Gating strategy for flow cytometry analysis. In murine blood samples, cells were first gated for singlets (FSC-H vs. FSC-A) and live cells (SSC-A vs. LIVE/DEAD aqua). The blasts were further gated based on the expression of hCD45 and hCD33, and absence of mCD45 expression. **(D)** Fold increase numbers of blasts quantified by FACS analysis in blood. For each mouse, the number of hCD45^+^ cells per microliter of blood at days 64, 74, and 81, was normalized to the number of hCD45^+^ cells before treatment (day 64).

To evaluate the cytotoxic effect of pomalidomide in AML, primary AML cells from 23 patients at diagnosis were cultured with pomalidomide for 48 h, harvested, and subjected to viability analysis by flow cytometry. Pomalidomide treatment slightly reduced the viability of leukemia cells in this 48 h culture (mean ± SEM, 59.61 ± 3.53% of live cells with pomalidomide *versus* 64.39 ± 3.78% with DMSO, *p* = 0.0024) (Figure 1A). In order to get insight into the ability of pomalidomide to interfere both with primary AML survival and expansion, we treated a primary AML (upn20) that was able to expand in immunodeficient mice. *In vitro* treatment with pomalidomide induced an increase of death compared to DMSO control treatment (40.3% of live cells with pomalidomide *versus* 59.8% with DMSO, Figure 1B). We further assessed antitumor efficacy of pomalidomide *in vivo* using NSG mice xenografted with human primary AML blasts. AML blasts proliferated and rapidly colonized the mice. The administration of pomalidomide for four consecutive days led to a significant decrease in the number of circulating AML blasts as shown by flow cytometry analysis. The number of circulating AML blasts in pomalidomide-treated animals was significantly reduced 4 days (mean ± SEM, 0.90 ± 0.14 cells per μL of blood (fold increase compared for each mouse to pre-treatment time point) versus 3.85 ± 1.21, *p* = 0.0023) and 11 days after injection of pomalidomide (mean ± SEM, 2.52 ± 0.82 versus 8.24 ± 3.26, *p* = 0.0379) compared to DMSO control group (Figures 1C,D). These data suggest that pomalidomide have an intrinsic capacity of inducing tumor cell death and is effective in controlling leukemia growth.

### Pomalidomide Sensitizes AML Blasts to NK Cell-Mediated Lysis

**Figure 2:**
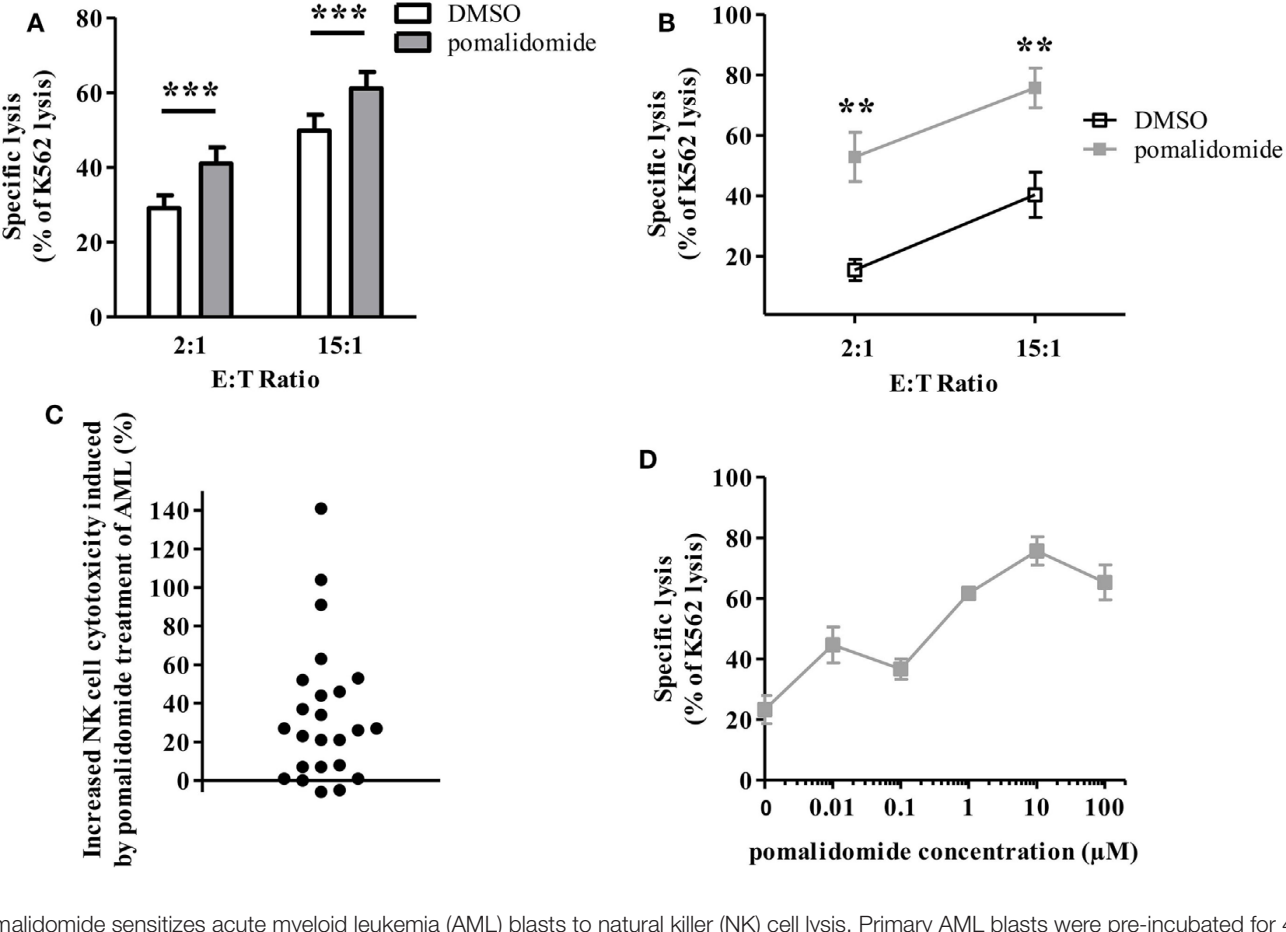
Pomalidomide sensitizes acute myeloid leukemia (AML) blasts to natural killer (NK) cell lysis. Primary AML blasts were pre-incubated for 48 h with pomalidomide (10 µM) or DMSO. They were extensively washed before the addition of allogeneic NK cells from healthy volunteers (HV). Standard [51Cr]-release assay was used to evaluate specific lysis by NK cells. Results were presented normalized to K562 lysis. **(A)** Comparison of the sensitivity of AML blasts (*n* = 24) after treatment with pomalidomide or DMSO to allogeneic NK cells (*n* = 2–12). Data are shown for E:T 2:1 and 15:1. **(B)** Representative data from one experiment: primary AML blasts (from patient upn20) lysis by NK cells from HV (*n* = 10). **(C)** For each AML patient (*n* = 24), the cytotoxicity by NK cells from healthy volunteers (*n* = 3–12) at E:T ratio 15:1 in the pomalidomide condition was evaluated relatively to the DMSO condition and represented as percent of increase of cytotoxicity. For each patients, the mean of [(specific lysis of AML treated by pomalidomide—specific lysis of AML treated by DMSO)/specific lysis of AML treated by DMSO] was represented. **(D)** Dose-response effect of pomalidomide on enhancement of AML blasts lysis by allogeneic NK cells (*n* = 3). Primary AML blasts from one patient were pre-incubated with 10-fold dilutions of pomalidomide starting from 100 µM, or DMSO as the control treatment. Results are expressed as mean ± SEM and statistical significance was established using paired Student’s *t*-test **(A)** and Wilcoxon matched pairs test **(B)**.

We next sought to determine whether IMiDs could increase the sensitivity of AML blasts to NK-cell mediated cytotoxicity. We thus determined whether AML cell sensitivity to NK cell-mediated killing was modified by pre-incubation of tumor cells with IMiDs. PBMCs from 24 AML patients at diagnosis, with more than 74% of blasts (mean 89%, range 74–98%), were cultured with pomalidomide for 48 h. Cells were thoroughly washed before use in cytotoxicity assays. NK cells sorted from PBMC from HV and IL-2 and IL-15 activated overnight were used as effector cells. The K562 cell line, a highly NK-sensitive cell line, was used as a positive control for NK cell cytotoxicity in a standard 4 h ^51^Cr release assay. Levels of AML cell lysis were standardized to the levels achieved against K562 cells. We found that treatment of tumor cells with pomalidomide prior to incubation with NK cells, significantly increased AML cell lysis (Figure 2A; Figure S1A in Supplementary Material, *p* < 0.0001, Figure 2B). Of note, lenalidomide and pomalidomide had similar effects (Figure S1B in Supplementary Material). Initially, primary AML blasts were differentially sensitive to allogeneic NK cell lysis as shown by Figure S1C in Supplementary Material. We also observed a gradation in the response to pomalidomide between patients (Figure 2C), which was not correlated with basal sensitivity to allogeneic NK cell lysis (data not shown). To preclude the impact of HLA/KIR-dependent alloreactivity on the increase of sensitivity of AML cells to NK cell lysis by pomalidomide, we have tested several allogeneic NK cells for each AML donor. Response to pomalidomide was similar irrespective of NK cells’ donor, as shown for upn20 in Figure 2B. This suggests that increased sensitivity of AML cells to NK cell lysis by pomalidomide is not dependent on KIR ligand mismatch.

Pomalidomide plasma concentration in HV is 0.05–0.38 µM depending on pharmacokinetics studies (Cmax 0.05–0.39 µM after a single 2-mg oral dose; Cmax 0.18 µM after a single 4-mg oral dose). *In vitro*, pomalidomide sensitized primary AML blasts to NK cell lysis with estimated concentration found in the plasma of patients, in a dose-dependent fashion (Figure 2D).

### Pomalidomide Pretreatment of Leukemia Cells Markedly Enhances NK Cell Functions

**Figure 3:**
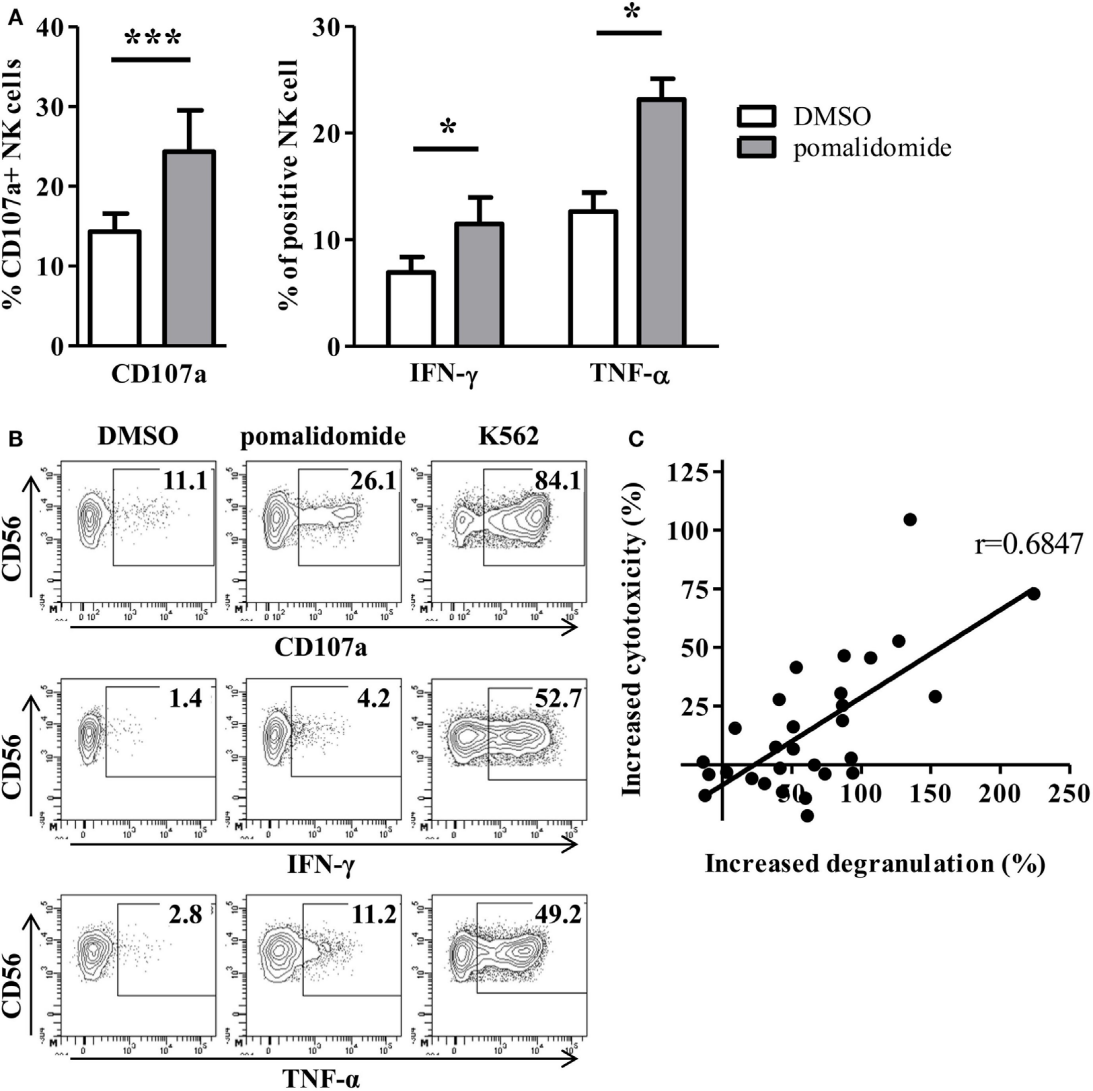
Pomalidomide pretreatment of leukemia cells markedly enhances natural killer (NK) cell functions. After 48 h incubation of primary acute myeloid leukemia (AML) blasts with pomalidomide or DMSO, the cells were extensively washed before the co-culture with NK cells from healthy volunteers (HV) for 4 h. NK cell functions were assessed by flow cytometry. **(A,B)** Percentage of HV-NK cells degranulating (CD107a^+^), and IFN-γ and TNF-α production, was monitored using cytometry. K562 cell line was used as a positive control. **(A)** Data obtained from NK cells (*n* = 1–5) in response to 12 primary AML blasts treated by pomalidomide or DMSO are presented. Results were expressed as mean ± SEM and statistical significance was established using Wilcoxon matched pairs test. **(B)** Representative dot plot from one experiment. **(C)** Linear correlation of the AML blasts (*n* = 12) sensitivity to pomalidomide (increased cytotoxicity by allogeneic NK cells due to AML pomalidomide treatment) and the NK cell degranulation (increased degranulation) (*n* = 1–5) correlation was established using Pearson’s correlation coefficient.

We next determined the effects of pomalidomide on NK effector cell functions in the model where target AML cells were pre-incubated with pomalidomide, and then co-incubated with purified NK cell from HV. Both NK cell degranulation and NK cell production of cytokines (IFN-γ and TNF-α) were enhanced (CD107a, *p* = 0.0010; IFN-γ, *p* = 0.0469; TNF-α, *p* = 0.0156, Figures 3A,B). Noteworthy, we observed that pomalidomide-mediated increased NK cell degranulation correlated with pomalidomide-mediated increased cytotoxicity (*r* = 0.6847) (Figure 3C). We also investigated the effects of pomalidomide on NK cells in a model where only NK cells were incubated in presence of pomalidomide. Pretreatment of purified NK cells with pomalidomide did not enhance cytotoxicity toward AML cells (Figure S2A in Supplementary Material) nor degranulation or cytokine production (Figure S2B in Supplementary Material). Finally, in a model where both primary AML cells and effector NK cells were pre-incubated separately with pomalidomide, the increased sensitivity of AML cells to NK cell lysis was similar as in the model where only AML cells were pre-incubated with pomalidomide (Figure S2C in Supplementary Material). Together, these data suggest that the increased sensitivity of AML cells to NK cell lysis by IMiDs is dependent on AML blasts and not on NK cells.

### IMiDs Induce NK Cell Phenotypic Modifications

**Figure 4:**
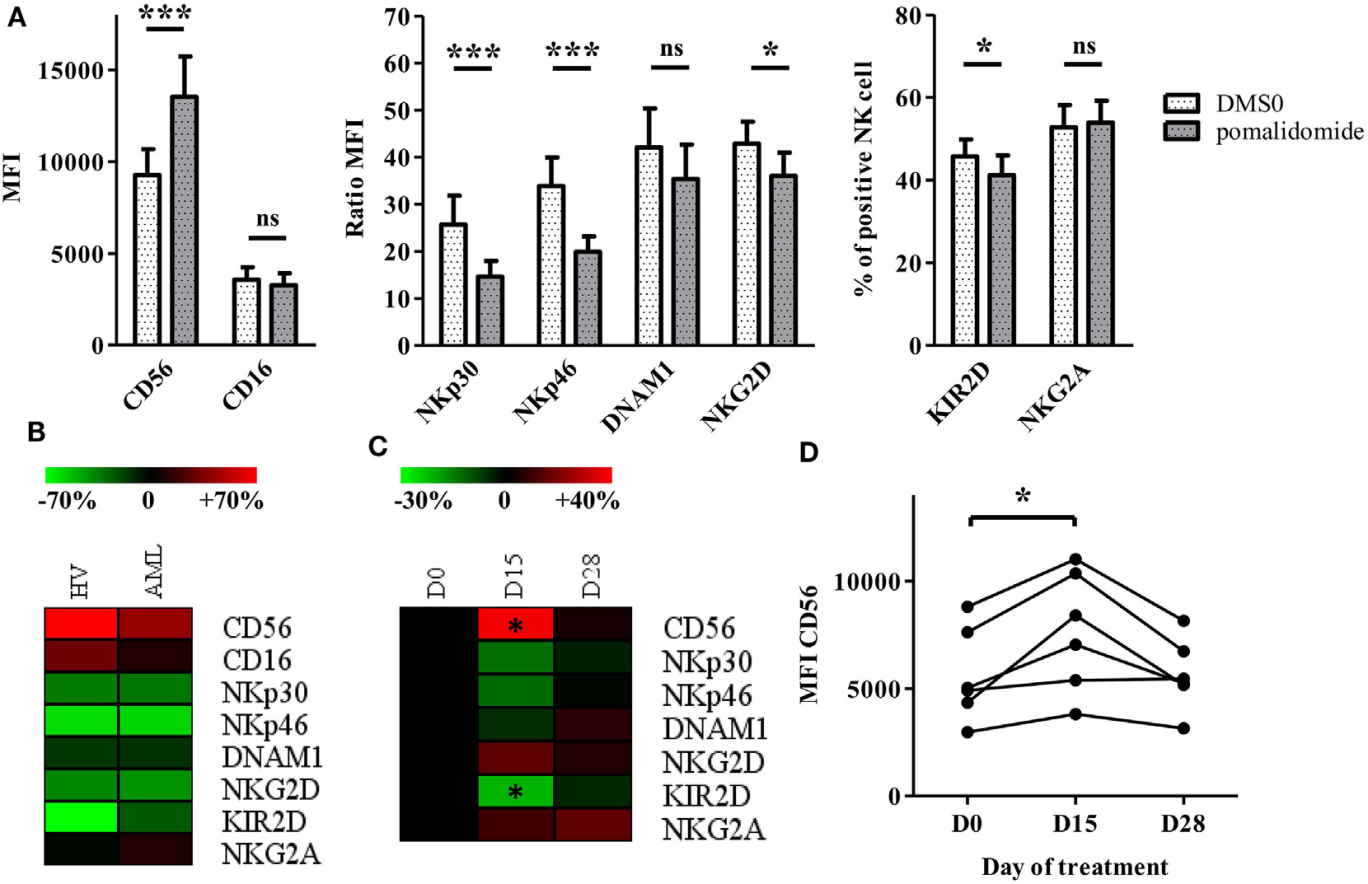
Immunomodulatory drugs induce natural killer (NK) cell phenotypic modifications *in vitro* and *in vivo*. **(A)** Purified NK from healthy volunteers (HV) (*n* = 13) were pre-incubated for 48 h with pomalidomide (10 µM) or DMSO. Surface NK-cell receptors were expressed as mean fluorescence intensity (MFI), ratio MFI or percent of positive NK cells. Results were expressed as mean ± SEM and statistical significance was established using Wilcoxon matched pairs test. **(B)** PBMCs from HV (*n* = 7) or acute myeloid leukemia (AML) patients (*n* = 5) were incubated with pomalidomide or DMSO. Surface NK-cell receptors in the pomalidomide condition were presented as the relative variation range from −70% (green) to +70% (red) relatively to the DMSO condition. **(C,D)** PBMCs from patients with myeloid malignancies treated with lenalidomide (*n* = 6) were analyzed by flow cytometry at day0 (D0), D15, and D28 for surface NK-cell receptors expression. Results were presented as the relative variation range from −30% to +40% relatively to D0 **(C)**. MFI for CD56 **(D)**. Statistical significance was established using Wilcoxon matched pairs test between D0 and D15.

We were interested in evaluating the phenotypic impact of IMiDs on NK cells. We used PBMCs from HV and AML patients at diagnosis and analyzed NK cells for expression of CD56 and activating and inhibitory receptors after *in vitro* pomalidomide exposure. In HV, pomalidomide induced upregulation of CD56 and downregulation of activating receptors NKp30, NKp46, and inhibitory receptors KIR2D (Figure 4A). Of note, lenalidomide treatment induced similar effects (data not shown). The treatment of PBMCs from AML patients with pomalidomide induced similar NK cell phenotypic modifications compared to HV’s NK cells (Figure 4B). To evaluate IMiDs effect *in vivo* on NK cells phenotype, we performed an immunomonitoring analysis of blood samples from patients with myeloid malignancies treated with lenalidomide on days 1–21 of repeated 28 day cycles. PBMCs from treated patients were analyzed at D0, D15, and D28 of a lenalidomide cycle. Lenalidomide induced upregulation of CD56 (*p* = 0.0313) and downregulation and KIR2D (*p* = 0.0313) on NK cells. Although not statistically significant, lenalidomide induced also a downregulation of NKP30 and NKP46. These phenotypic modifications were restored at d28, after the 7 day rest period (Figures 4C,D). Collectively, these data revealed effects of IMiDs on NK cell phenotype, consistent *in vitro* and *in vivo*.

### Pomalidomide Downregulates HLA-Class I on Primary AML Cells *In Vitro*

**Figure 5:**
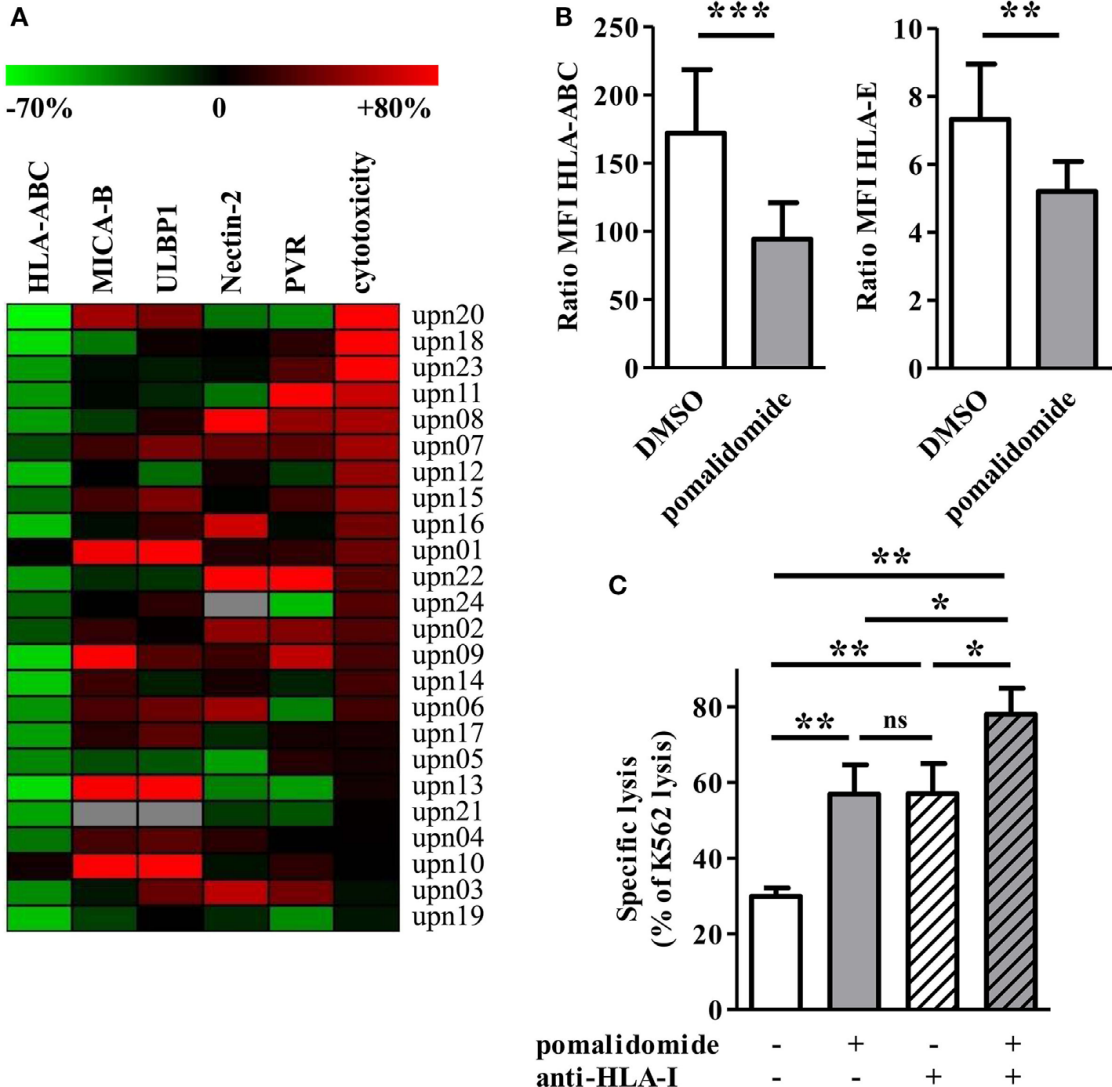
Pomalidomide induces downregulation of MHC class I and change of the expression of natural killer (NK) cell ligands on acute myeloid leukemia (AML) blasts. **(A)** The expression of surface markers and cytotoxicity in the pomalidomide condition are represented relatively to the DMSO condition. The relative variation ranges from −70% (green) to +80% (red). The heatmap is sorted according the cytotoxicity level (rightmost column). HLA-ABC is clearly decreased for the majority of the samples. **(B)** The expression of HLA-ABC and HLA-E are represented in the DMSO and pomalidomide conditions on the left and right graphics, respectively (*n* = 24). Results were expressed as mean ± SEM and statistical significance was established using Wilcoxon matched pairs test. **(C)** Comparison of the sensitivity of AML blasts (*n* = 3) to allogeneic NK cells (*n* = 2–4), after treatment of AML blasts with pomalidomide or DMSO control and then incubation with anti-HLA-I-blocking mAbs or isotype control mAbs. Data are shown for E:T 15:1. Results were expressed as mean ± SEM and statistical significance was established using Wilcoxon matched pairs test.

Acute myeloid leukemia cells are susceptible to NK-mediated killing. Engagement of several ligands is implicated in AML cell recognition and activation of NK cells. Therefore, we analyzed primary AML blasts for expression of ligands for NK cell activating receptors and assessed the impact of pomalidomide treatment on their surface expression. MICA-B, ULBP1 (ligands for NKG2D), Nectin-2, and PVR (ligands for DNAM-1) were expressed by primary AML blasts (Figure S3A in Supplementary Material). AML cells exposed to pomalidomide displayed an alteration of the expression of activating ligands. However, alteration of expression varied among the patients. These variable phenotypic modifications on primary AML blasts induced by pomalidomide were not associated with increased lysis by NK cells (Figure 5A). Of note, the level of CD45 expressed on primary AML cells remained constant after treatment with pomalidomide (data not shown).

We next studied pomalidomide effect on HLA-class I level on AML cells. Flow cytometry revealed an important downregulation of HLA-ABC after pomalidomide treatment of AML blasts (39.3 ± 3.7% decrease, *p* < 0.0001, Figures 5A,B). HLA-ABC downregulation was also observed on pomalidomide-treated AML cell lines. Pomalidomide reduced HLA-ABC expression in a dose- and time-dependent fashion on the MOLM-14 and HL-60 cell lines and primary AML cells (Figures S3B,C in Supplementary Material). Of note, HLA-E was also downregulated by pomalidomide treatment (*p* = 0.0066, Figure 5B). There was no correlation between the variation of cytotoxicity and the variation of HLA-ABC downregulation induced by pomalidomide (*r* = 0.3172, Figure S3D in Supplementary Material). These data suggest that HLA down-modulation by pomalidomide may not be the only mechanism explaining the enhanced sensitivity to NK cells.

To address the functional consequence of pomalidomide treatment and HLA-I blockade on AML cells, the cytotoxicity of NK cells was measured against AML-cell targets pretreated with DMSO or pomalidomide and then incubated in the presence or absence of anti-HLA-I blocking mAbs. As expected, NK-cell cytotoxicity was enhanced in the presence of anti-HLA-I blocking antibody. Hence, the blockade of HLA-I resulted in a similar enhancement of NK-cell cytotoxicity compared to pomalidomide-treated AML cells (Figure 5C). Blocking HLA-I in addition to pomalidomide treatment of AML cells resulted in an even higher cytotoxicity, revealing an additive effect.

### Cereblon Expression Between AML Cells Is Not Involved in the Pomalidomide-Mediated Sensitization of AML to NK Activation

**Figure 6:**
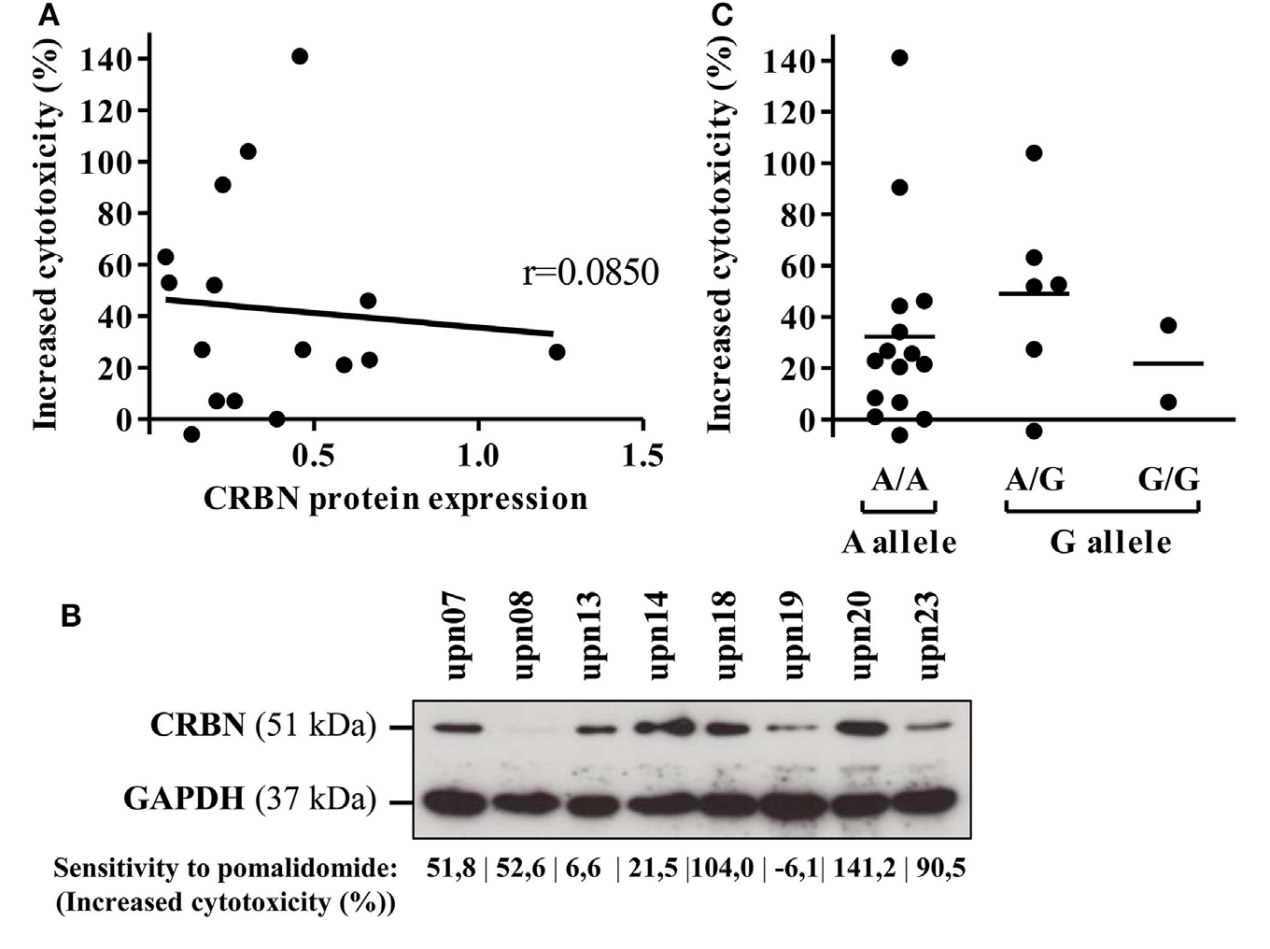
Cereblon (CRBN) mRNA, protein levels, and polymorphism do not correlate in acute myeloid leukemia (AML) samples with different sensitivity to pomalidomide. Primary AML blasts express CRBN irrespectively of their sensitivity to pomalidomide. **(A)** Linear regression of the AML blast sensitivity to pomalidomide (increased cytotoxicity by allogeneic natural killer cell) and CRBN protein expression (normalized to GAPDH) evaluated by western blot (*n* = 16). Correlation was established using Pearson’s correlation coefficient. **(B)** Western blot analysis of total protein extracts from primary AML blasts poorly or well sensitized by pomalidomide. **(C)** Genotyping analysis for A/G CRBN polymorphism for primary AML cells (*n* = 23).

Cereblon is the only identified target of IMiDs. Therefore, we analyzed CRBN protein and mRNA expression on primary AML blasts. CRBN protein expression was heterogeneously detected in all 16 investigated samples (Figure 6A). However, high expression of CRBN was not associated with improved pomalidomide-mediated sensitization of AML to NK lysis (*r* = 0.0850). For instance, two (upn08 and upn23) out of five AML cells highly sensitive to pomalidomide had low CRBN protein expression; the three other patients (upn07, upn18, and upn20) had blasts with high levels of CRBN protein, suggesting that the origin of pomalidomide sensitivity of these cells is unlikely due to a high expression of CRBN protein (Figure 6B).

Cereblon expression of AML leukemia cells was also confirmed at the mRNA level by qRT-PCR. Similarly, we did not observed significant association between CRBN mRNA levels with the sensitivity of primary AML blasts to pomalidomide (Figure S4 in Supplementary Material).

Finally, we investigated A/G polymorphism, located at the site 29 nucleotides before the transcriptional start site of the CRBN gene. In our cohort, the distribution of A and G alleles (65.2 and 34.8%, respectively) was not statistically different (*p* = 0.095, chi-quare test) from that of the general population as described (75.3 and 24.7%) in genome.ucsc.edu. A/G polymorphism of the CRBN gene did not correlate with the sensitivity of primary AML blasts to pomalidomide (Figure 6C).

Altogether, these data revealed that neither CRBN expression nor A/G polymorphism is associated with the pomalidomide sensitization of primary AML blasts to NK cell lysis.

## Discussion

Immunomodulatory drugs are anticancer drugs widely used for the treatment of hematologic malignancies including MM and MDS. Mechanisms of action of IMiDs are not completely known but involve immunomodulation of immune cells including NK cells. NK cells are anti-leukemic cells particularly in the context of AML, where their activity is impaired (31, 40, 52–54). We therefore studied the impact of IMiDs on survival of primary AML blasts and on AML sensitivity to NK cell recognition. We have shown that IMiDs exert a direct cytotoxicity against primary AML cells and increase the sensitivity of primary AML cells to NK cell-mediated lysis.

Our data show that pomalidomide is capable of inducing death in leukemia cells *in vitro*. In addition, we have shown that pomalidomide has an antitumor activity *in vivo* in a NSG xenograft model. Interestingly, pomalidomide treatment led to a significant decrease in tumor progression. Thus, our data show that it is possible to establish a mouse model for testing IMiDs *in vivo*, and it strongly suggests that pomalidomide could be a powerful tool for AML treatment. Previous data obtained on hematologic cancer cells have revealed that IMiDs present anti proliferative and pro-apoptotic activities. First, lenalidomide inhibit proliferation in CLL, MM and lymphoma by causing cell cycle arrest p21^WAF-1^-dependent (7, 9, 55). Second, IMiDs induce apoptosis by caspase activation in MM and BPDCN (Blastic Plasmacytoid Dendritic Cell Neoplasm) cells [(10); K (56, 57)]. The exact signaling events occurring following pomalidomide exposure to sensitize AML cells are yet to be determined. Plausible hypothesis could be an activation of apoptosis in leukemia cells. Moreover, using NSG mice, our data suggest that the action of pomalidomide is independent of NK, T, and B cells, although we cannot exclude a mechanism implicating macrophages (58).

In addition to the direct anti-survival or impairment of AML progression of pomalidomide, we showed for the first time that IMiDs pre-treatment of primary AML cells, increased AML cell killing by allogeneic NK cells. Importantly, we observed a similar increase of AML cell killing by NK cells with lenalidomide and pomalidomide. Moreover, this observation was made with concentrations as low as 0.01 µM, below the 0.05 µM estimated concentration found in the plasma of patients treated daily with 2 mg of pomalidomide (59, 60). We also showed that this effect was dependent on AML cell treatment and not NK cell treatment. The increased sensitivity by pomalidomide of AML cells to NK cell lysis was found with all NK donors, precluding a KIR-HLA dependency.

In addition, the pomalidomide pre-treatment of AML cells also led to an enhancement of other effector function of NK cells, such as IFN-γ and TNF-α production. Using AML cell lines, pretreatment with lenalidomide has been recently shown to improve the polarization rate of lytic granule in the NK-AML immunological synapse (51). Hence, target cell-mediated degranulation is correlated to a strong polarization of the effector machinery in NK cells (61). By evaluating degranulation by cytometry, our data (on primary leukemic cells) indeed suggested that pomalidomide treatment of AML blasts might improve the polarization of the synapse between AML cells and NK cells.

When studying IMiDs effects on NK cells, we found that purified NK cell treatment with IMiDs did not alter the NK cell functions and lysis of primary allogeneic AML blasts.

Altogether, these data suggested that IMiDs sensitization of AML blasts to NK cell functions is mostly dependent on AML and not on NK cells.

Our investigation of IMiDs effect on NK cell phenotype revealed that IMiDs induced upregulation of CD56 and downregulation of NKp30, NKp46, and KIR2D on NK cells from AML patients and HV *in vitro*. The phenotypic changes observed with IMiDs are in agreement with previously reported by Dauguet et al., for lenalidomide (19). Furthermore, in a cohort of patients with myeloid malignances treated with lenalidomide, we observed similar phenotypic modifications on NK cells after IMiDs *in vivo* treatment. Interestingly, NK phenotypic changes were restored after the 7 day rest period in patient treated once daily on days 1–21 of repeated 28 day cycles, suggested that lenalidomide have a rapid and transient effect on NK cell phenotype on patients. In contrast to the Lioznov study, we observed a downregulation of NCRs. These discordances could be explained by different time collection. Lioznov and col investigated lenalidomide long-term effects, whereas we looked for effects during a cycle of treatment (18). This suggests that lenalidomide has different short-term and long-term effects on NK cell phenotyping. A larger cohort is needed to confirm these phenotypic changes, in particular on NCRs expression. Finally, as we have shown a strong upregulation of CD56 on NK cells during a lenalidomide cycle, we suggest the monitoring of the increased CD56 surface expression on NK cells as a surrogate marker for pomalidomide’s impregnation.

Natural killer cell alloreactivity is dependent on a repertoire of activating and inhibitory receptors. AML cells express ligands for NK-cell activating receptors, making them potential targets for NK cell lysis (62, 63). We investigated pomalidomide impact on AML phenotype, especially the key ligands involved in AML cell recognition by NK cells, including ligands for DNAM-1 and NKG2D. We showed in this study that pomalidomide treatment of primary AML blasts altered surface expression of ligands for activating NK receptors. Prior study on primary MM cells showed that lenalidomide enhances expression of ULBP-1 (64). We observed in AML that pomalidomide induced phenotypic modifications of NK cell activating ligands including ULBP-1, but not reproducibly between AML patients’ samples. AML is a heterogeneous disease in terms of genetic and outcome. Hence, it is therefore conceivable to observe some degree of heterogeneity in terms of drug response.

We also investigated pomalidomide impact on ligands for NK inhibitory receptors, expressed by AML cells. Pomalidomide downregulated HLA class I on AML cells, including HLA-ABC and HLA-E. As expected, the use of HLA-I blockade antibody augmented NK-cell mediated killing of primary AML blasts (45, 64). Interestingly, pomalidomide pre-treatment of AML blasts improved the HLA-I blockade effect. Our findings encourage continuing investigation of pomalidomide treatment in association to the blockade of KIR/HLA or NKG2A/HLA-E interactions by therapeutic antibodies in AML.

To date, the presence of a deletion 5q with or without other chromosomal abnormalities is the best response prognosis factor of lenalidomide treatment in myeloid malignancies patients (MDS syndrome and AML). Moreover, high expression of CRBN, the only known molecular target of IMiDs, was recently associated with improved clinical response in patients with MM treated with lenalidomide and dexamethasone (25). Previously, it has been shown that IMiDs antiproliferative activity in myeloma and CLL was dependent on CRBN (9, 22, 23). Conversely, Gandhi et al. revealed a lack of correlation between CRBN mRNA or protein levels with the antiproliferative sensitivity of myeloma cells to IMiDs (65). Although, primary AML cells expressed CRBN, we showed that there was no correlation between CRBN expression and the sensitivity of primary AML to pomalidomide. High expression of CRBN was neither associated with pomalidomide capacity of inducing AML cell death, nor with pomalidomide increased sensitivity of AML blasts to NK cell lysis. Importantly, A/G polymorphism has been identified at the site 29 nucleotides before the transcriptional start site of the CRBN gene. In MDS, Sardnal et al. observed a higher distribution of G allele as a biomarker of lenalidomide response in low/int-1-risk MDS without del(5q) (26). In AML, G allele content was not associated with the cytotoxic effect of pomalidomide on AML cells. Collectively, these data suggest that pomalidomide sensitivity of AML cells could not be explain by CRBN expression or A/G polymorphism. However, as suggested by the recent study of Dimopoulos et al., in myeloma, resistance to IMiDs could be associated with epigenetic events that induce a reduction in gene expression (66). Epigenetic mechanisms such as DNA methylation and chromatin accessibility could be interesting to investigate in AML response to IMiDs. Furthermore, the study of Fischer et al. suggests that evaluating endogenous substrates of CRBN such as MEIS2 and neo-substrates such as Ikaros and Aiolos may be considered as potential mechanisms of AML sensitivity to IMiDs (67).

Overall, we provide the first evidence that IMiDs have an antitumor activity in AML by inducing cell death in leukemic cells and increasing their sensitivity to NK cell-mediated lysis. Of note, although IMiDs induce NK cell phenotypic modification, functionality of NK cells is preserved. Our data open new perspectives for immunotherapy in AML by targeting blasts, directly or *via* NK cell-mediated immune activation. In particular, IMiDs induced downregulation of HLA-class I on AML blasts suggests that combination of IMiDs with strategies blocking KIR/HLA or NKG2A/HLA-E interactions by therapeutic antibodies is encouraging. Moreover, combining IMiDs and therapy-inducing ligands expression for NK-activating receptors (68) could be attractive.

## Ethics Statement

Samples for the study were collected from patients from the Institut Paoli-Calmettes after informed consent obtained from all participants in accordance with the Declaration of Helsinki. For the immunomonitoring study, patients from the Institut Paoli-Calmettes, were prospectively recruited between January 2012 and December 2013. The study number 2012-A01381-42 was undertaken in accordance with the principles of the Declaration of Helsinki and Good practice guidelines and after local ethics committee approval. Each patient gave written informed consent. Mice maintenance and experimental procedures were performed in accordance with protocols approved and compliance with policies approved by the local Committee for Animal Experimentation of Marseille (CAE of Provence number 14), France (2-091009).

## Author Contributions

AR designed the research, performed the experimental work, analyzed and interpreted the data, and drafted the paper; AG, FR, AB, CC, FO, AB-A, and FP performed the experimental work and analyzed the data; RC and SG designed the research, performed the experimental work, analyzed and interpreted the data, and contributed to draft the paper; CF contributed to draft the paper; TB, J-JF, YC, J-LM, and NV contributed to the design of the project research; DO designed the research and contributed to the analysis and interpretation of data and to draft the paper.

## Conflict of Interest Statement

The authors declare that the research was conducted in the absence of any commercial or financial relationships that could be construed as a potential conflict of interest. The reviewer KT and handling Editor declared their shared affiliation.
