# Supplementary material for: Immunomodulatory Drugs Exert Anti-Leukemia Effects in Acute Myeloid Leukemia by Direct and Immunostimulatory Activities
Source: Front Immunol. 2018 May 4;9:977. doi: 10.3389/fimmu.2018.00977 (PMC5945824; doi:10.3389/fimmu.2018.00977)
Supplement: Supplementary file 1 [file Presentation_1.PDF]

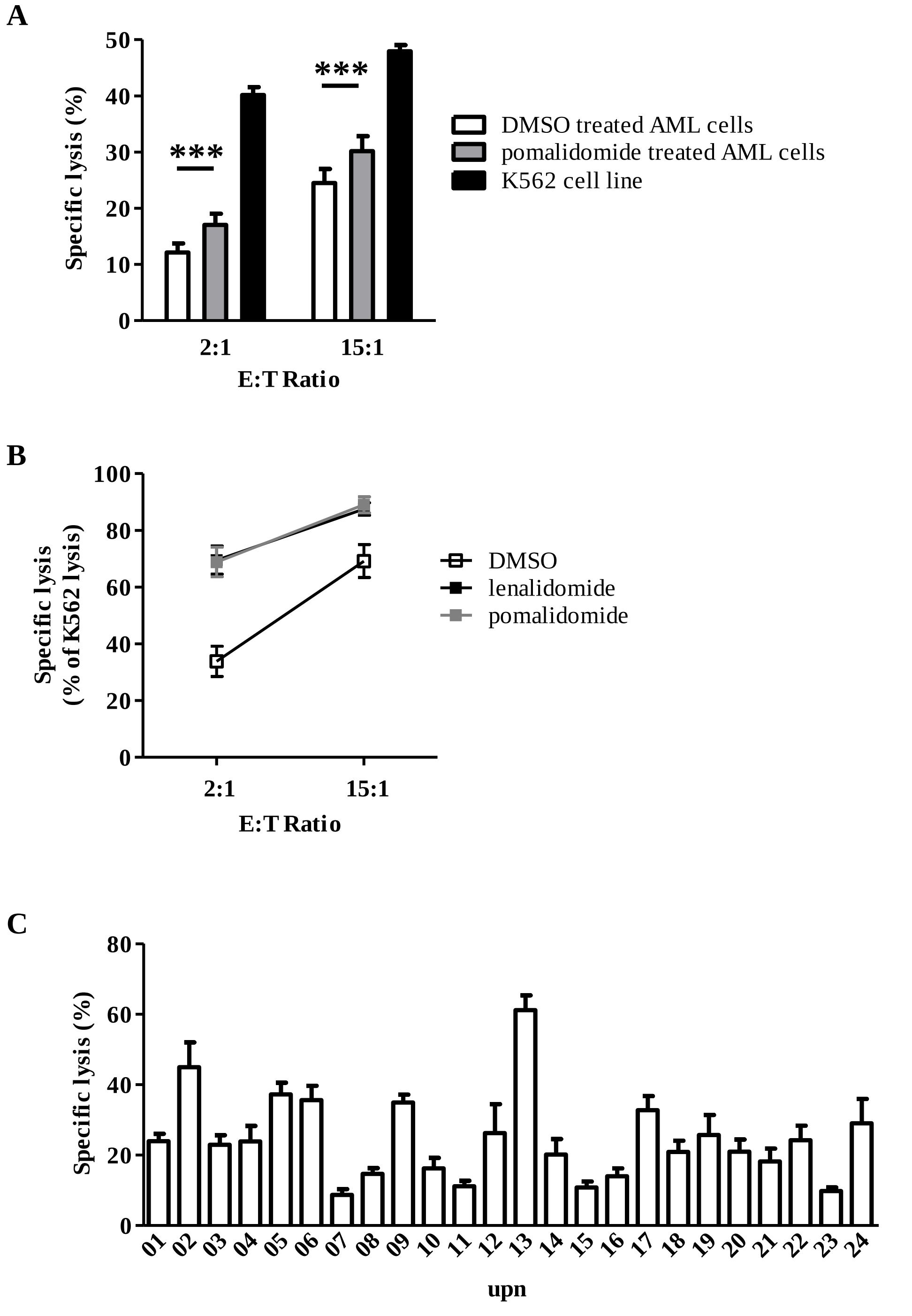


**Supplementary Figure 1.** Pomalidomide sensitizes AML blasts to NK cell lysis. (A and B) NK-mediated lysis of K562 cells and AML blasts (*n=24*) after treatment with (A) pomalidomide or DMSO control, or (B) pomalidomide, lenalidomide or DMSO. Data are shown for E:T 2:1 and 15:1. (C) Sensitivity of blasts from 24 AML patients to lysis by allogeneic NK cell *(n=2-12)* (E:T 15:1). Results are expressed as mean±sem and statistical significance was established using paired Student *t* test (A).

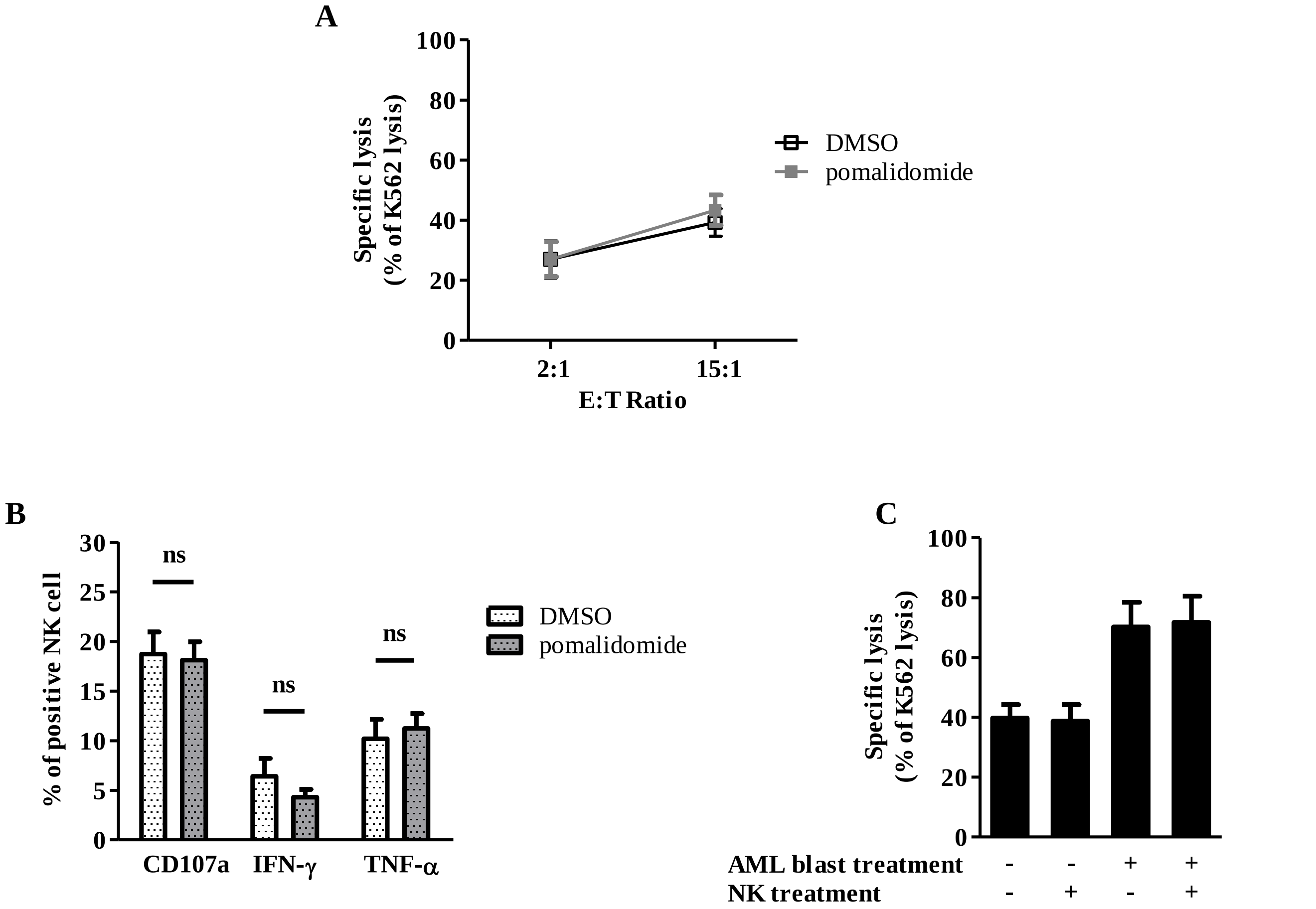


**Supplementary Figure 2.** Effect of IMiDs on NK cell phenotype and functions. (A-C) Purified NK from HV were pre-incubated for 48 hours with pomalidomide (10 µM) or DMSO. (A) Standard [$^{51}$Cr]-release assay was used to evaluate specific lysis of allogeneic NK cells *(n=4)* against primary AML blasts (upn20). (B) Percentage of NK cells *(n=2-3)* degranulation (CD107a), and IFN-γ and TNF-α production, in response to AML cells *(n=12)* was monitored using cytometry. (C) Primary AML blasts from one patient and allogeneic NK from 3 HV were pre-incubated separately for 48 hours with pomalidomide (+) (10 µM) or DMSO (-) as control treatment. They were extensively washed before the co-culture. Standard [$^{51}$Cr]-release assay was used to evaluate specific lysis. Results were expressed as mean±sem and statistical significance was established using Wilcoxon matched-pairs test.

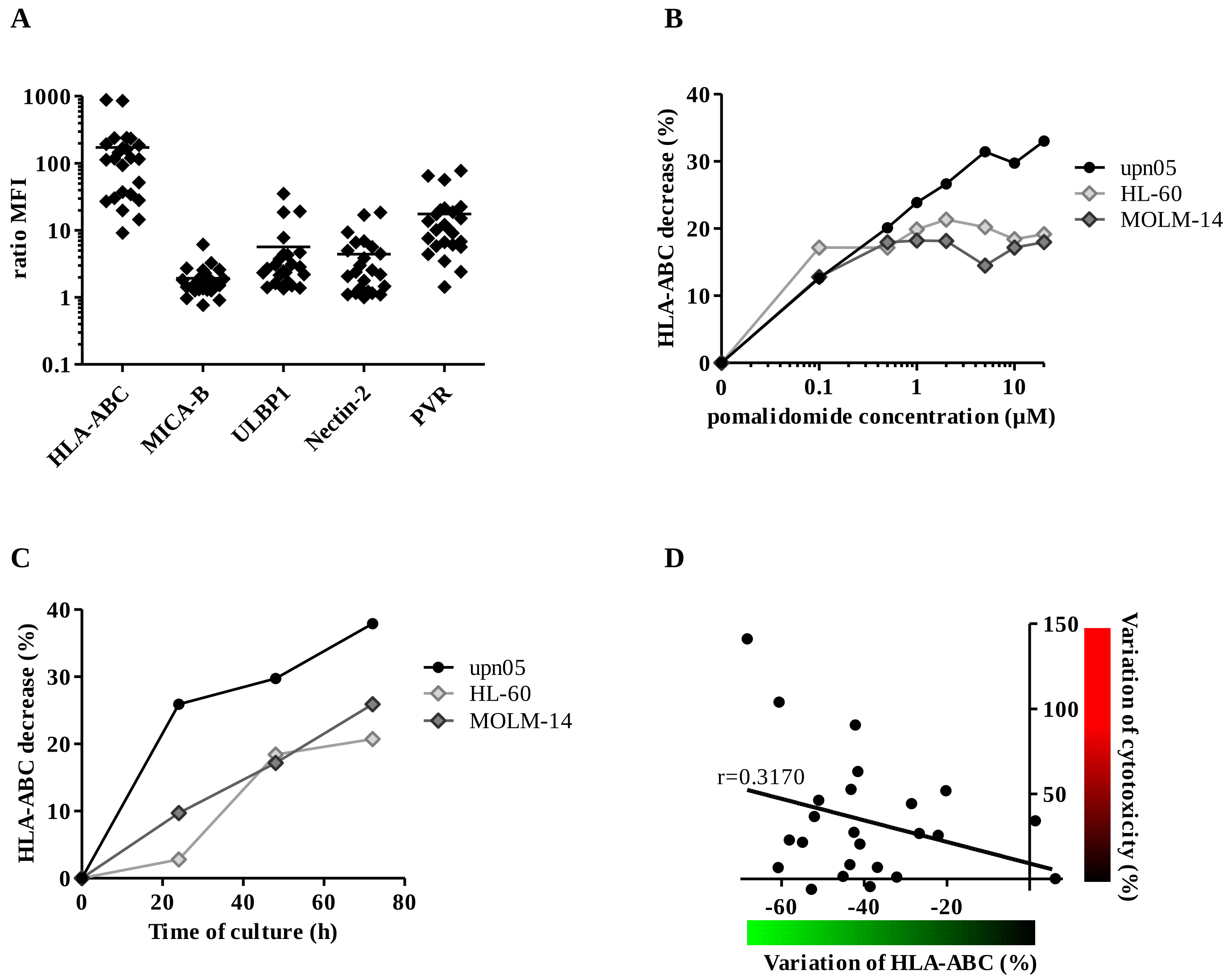


**Supplementary Figure 3.** Pomalidomide-induced down-regulation of MHC class I and change of the expression of NK cell ligands on AML blasts. (A) Expression levels of NK cell ligands for activating receptors (MICA, ULBP1, Nectin-2 (CD112) and PVR (CD155)) and inhibitory receptors (HLA-ABC), expressed as the ratio of the mean fluorescence intensity (MFI) for each marker divided by the MFI for the isotype control, were determined on primary AML blasts *(n=24)* by flow cytometry. (B and C) HLA-ABC downregulation (%) induced by pomalidomide on AML cell lines (HL-60 and MOLM-14) and primary AML blasts from one patient (upn05), was presented as described in Fig. 4 and evaluated in a (B) dose and (C) time-manner using flow cytometry. (D) The relative variation of cytotoxicity is correlated with the relative variation of HLA-ABC for each patient *(n=24)*.

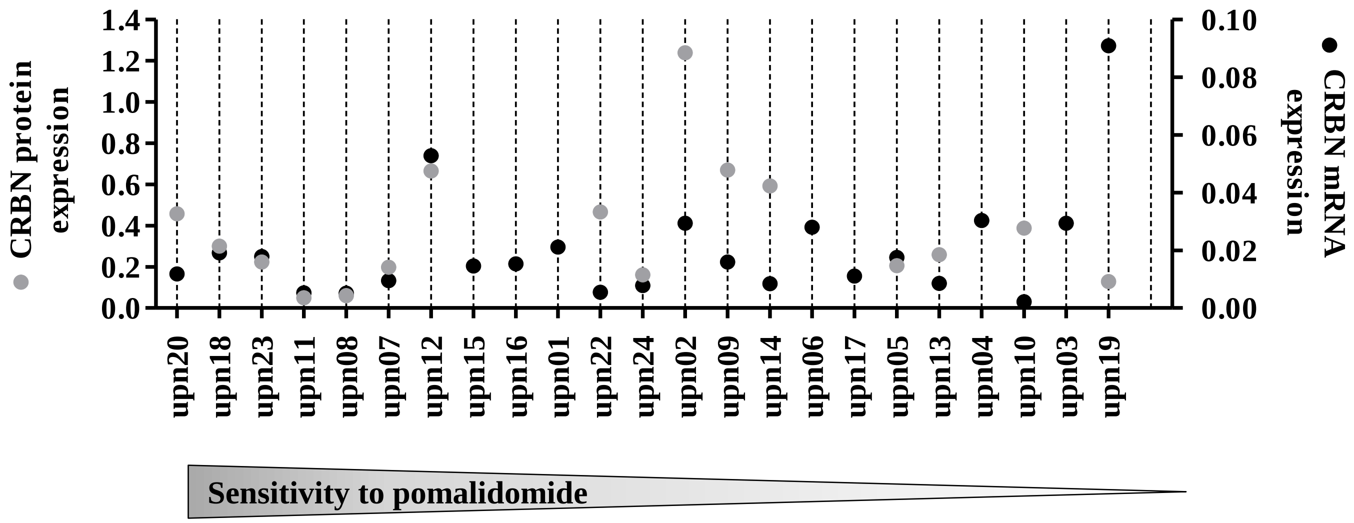


**Supplementary Figure 4.** CRBN protein and mRNA expression (normalized to GAPDH) from primary AML blasts *(n=23)*. CRBN protein expression was evaluated by western-blot. CRBN mRNA expression was evaluated by qRT-PCR and presented as a ratio $R = 2^{-\Delta Ct}$. AML blasts are classified according to a gradient of sensitivity to pomalidomide as described in Fig.2C (increased AML lysis by NK cell induced by pomalidomide treatment).
